# Combined epigenetic and immunotherapy for blastic and classical mantle cell lymphoma

**DOI:** 10.18632/oncotarget.28258

**Published:** 2022-08-16

**Authors:** Francis R. LeBlanc, Zainul S. Hasanali, August Stuart, Sara Shimko, Kamal Sharma, Violetta V. Leshchenko, Samir Parekh, Haiqing Fu, Ya Zhang, Melvenia M. Martin, Mark Kester, Todd Fox, Jiangang Liao, Thomas P. Loughran, Juanita Evans, Jeffrey J. Pu, Stephen E. Spurgeon, Mirit I. Aladjem, Elliot M. Epner

**Affiliations:** ^1^Department of Medicine, Pennsylvania State University College of Medicine and Penn State Hershey Cancer Institute, Hershey, PA 17033, USA; ^2^Department of Hematology/Oncology, Penn State Hershey Cancer Institute, Hershey, PA 17033, USA; ^3^BayCare Medical Group, Cassidy Cancer Center, Winter Haven, FL 33881, USA; ^4^Division of Hematology and Medical Oncology, Icahn School of Medicine at Mount Sinai, New York, NY 10029, USA; ^5^Developmental Therapeutics Branch, Center for Cancer Research, NCI, NIH, Bethesda, MD 20892, USA; ^6^Department of Pharmacology, University of Virginia, Charlottesville, VA 22908, USA; ^7^Department of Public Health Sciences, The Pennsylvania State University College of Medicine, Hershey, PA 17033, USA; ^8^Department of Medicine/Hematology-Oncology, UVA Cancer Center, Charlottesville, VA 22908, USA; ^9^Department of Anatomic Pathology, Pennsylvania State University College of Medicine, Hershey, PA 17033, USA; ^10^Department of Medicine and Cancer Center, University of Arizona College of Medicine, Tucson, AZ 85724, USA; ^11^Department of Medicine, Oregon Health and Science University, Portland, OR 97239, USA; ^12^Beverly Hills Cancer Center, Beverly Hills, CA 90211, USA; ^*^Co-first authors

**Keywords:** epigenetics, blastic mantle cell lymphoma, cladribine

## Abstract

Classical MCL (cMCL) constitutes 6–8% of all B cell NHL. Despite recent advances, MCL is incurable except with allogeneic stem cell transplant. Blastic mantle cell lymphoma (bMCL) is a rarer subtype of cMCL associated with an aggressive clinical course and poor treatment response, frequent relapse and poor outcomes. We treated 13 bMCL patients with combined epigenetic and immunotherapy treatment consisting of vorinostat, cladribine and rituximab (SCR). We report an increased OS greater than 40 months with several patients maintaining durable remissions without relapse for longer than 5 years. This is remarkably better then current treatment regimens which in bMCL range from 14.5-24 months with conventional chemotherapy regimens. We demonstrate that the G/A870 *CCND1* polymorphism is predictive of blastic disease, nuclear localization of cyclinD1 and response to SCR therapy. The major resistance mechanisms to SCR therapy are loss of CD20 expression and evasion of treatment by sanctuary in the CNS. These data indicate that administration of epigenetic agents improves efficacy of anti-CD20 immunotherapies. This approach is promising in the treatment of MCL and potentially other previously treatment refractory cancers.

## INTRODUCTION

Mantle cell lymphoma (MCL) is an incurable, rare B cell non Hodgkins lymphoma (NHL) characterized by the t(11;14) translocation and subsequent epigenetic dysregulation and overexpression of cyclin D1 through juxtaposition of IgH control elements [1–3]. Epigenetic dysregulation of cyclin D1, which is not expressed in normal B cells, perturbes the cell cycle, leading to immortalization and lymphomagenesis [4]. MCL can be subdivided into three major subtypes differentiated by aggressiveness, MIPI score, Ki67 proliferative index and overall survival (OS): Classical, indolent and blastic. *TP53* mutations are associated with aggressive cases with poor outcomes. The role of other biomarkers and mutations, such as *SOX11*, are still under investigation [5–7]. The blastic variant, classically recognized by its cell morphology and high proliferation rate, is seen as extremely aggressive and difficult to treat as evidenced by overall survival around 14.5 months with conventional chemotherapy regimens without autologous or allogeneic stem cell transplantation [8]. A more recent study found that bMCL and high MIPI score MCL patients had median OS of 24 months [9].

The landscape of treatment options for MCL is changing [10]. Many recent therapeutic advances have been made using treatment regimens based on aggressive chemotherapy, novel drug combinations, maintenance therapy and hematopoietic bone marrow stem cell transplant (BMT) [10, 11]. Progress is being made against this disease; however, although survival is improving, relapse remains common, especially in those with advanced and aggressive disease. The majority of improvement for this disease has been in patients younger than 65 [11, 12]. These young patients tolerate more aggressive treatment approaches and are better candidates for bone marrow transplant. Unfortunately, the majority of MCL patients are older and have comorbidities that disqualify them from these aggressive therapies.

The mainstay of therapy for older patients who are not eligible for aggressive therapies, such as BMT, is combination chemo- and immunotherapy. Rituximab maintenance immunotherapy has a survival benefit as well [13]. More recently, bendamustine plus rituximab (BR), R-CHOP (rituximab, cyclophosphamide, doxorubicin, vincristine, prednisone) and modified R-HyperCVAD (rituximab, cyclophosphamide, vincristine, doxorubicin, dexamethasone) have gained popularity but continue to have suboptimal complete response (CR) rates of 25–50% [14]. In an attempt to enhance these treatments, additional agents, such as bortezemib, bendamustine and cytarabine, have been added to existing regimens with measurable efficacy [15, 16]. The successes of small molecules with anti-CD20 therapies such as rituximab, ofatumumab and obinotuzumab in high-risk patients have led to investigation of agents with other unique mechanisms of action such as lenalidomide and ibrutinib [17, 18].

We and others have demonstrated that cladribine, a purine analogue with hypomethylating properties, has activity against MCL, especially in combination with anti-CD20 therapies such as rituximab and ofatumumab [19–23]. Previously we have shown that cladribine in combination with alemtuzumab could be used to overcome alemtuzumab resistance in T cell prolymphocytic leukemia [24]. Similarly, we found that these epigenetic drugs can overcome rituximab resistance in relapsed MCL patients [24]. Cladribine plus rituximab combination therapy has promising activity, especially in treatment naïve patients, and is a treatment option for treatment naïve elderly MCL patients [21, 25, 26]. Because cladribine has unique epigenetic activity that can inhibit both DNA and histone methylation, synergy with a histone deacetylase inhibitor (HDACi), vorinostat, was explored to determine if combination epigenetic agents could increase efficacy of anti-CD20 therapies. Relapsed and naïve MCL patients were treated with vorinostat (SAHA), cladribine and rituximab (SCR) regimen and followed for OS, progression free survival (PFS) and with correlative basic science studies to investigate potential mechanisms of action of this epigenetic/immunotherapy combination. The treatment of MCL with SCR in a recent Phase 1–2 trial resulted in high CR rates, which were found to be durable. The addition of vorinostat did not appear to lead to increased toxicity. Epigenetic therapy in MCL including the SCR regimen is worthy of additional study [22].

Using samples obtained from this trial, we performed correlative studies on the MCL patient population and focused on the clinical results of the most aggressive blastic MCL subset of patients. Here we report the characteristics, results and data to support antibody dependent cellular cytotoxicity (ADCC) and apoptosis as the major mechanisms responsible for the effectiveness of SCR therapy. Additionally, with the goal of stratifying patients by predictive markers for outcomes, starting with known markers, including cyclin D1 the main driver in MCL, the G/A870 cyclin D1 polymorphism, relevant gene expression profiles such as *Sox11* and *TP53* after treatment and anti-CD20 maintenance therapy were correlated with treatment response in MCL [19, 20].

## RESULTS

### Cladribine inhibits both DNA and histone methyltransferases

**Figure 1:**
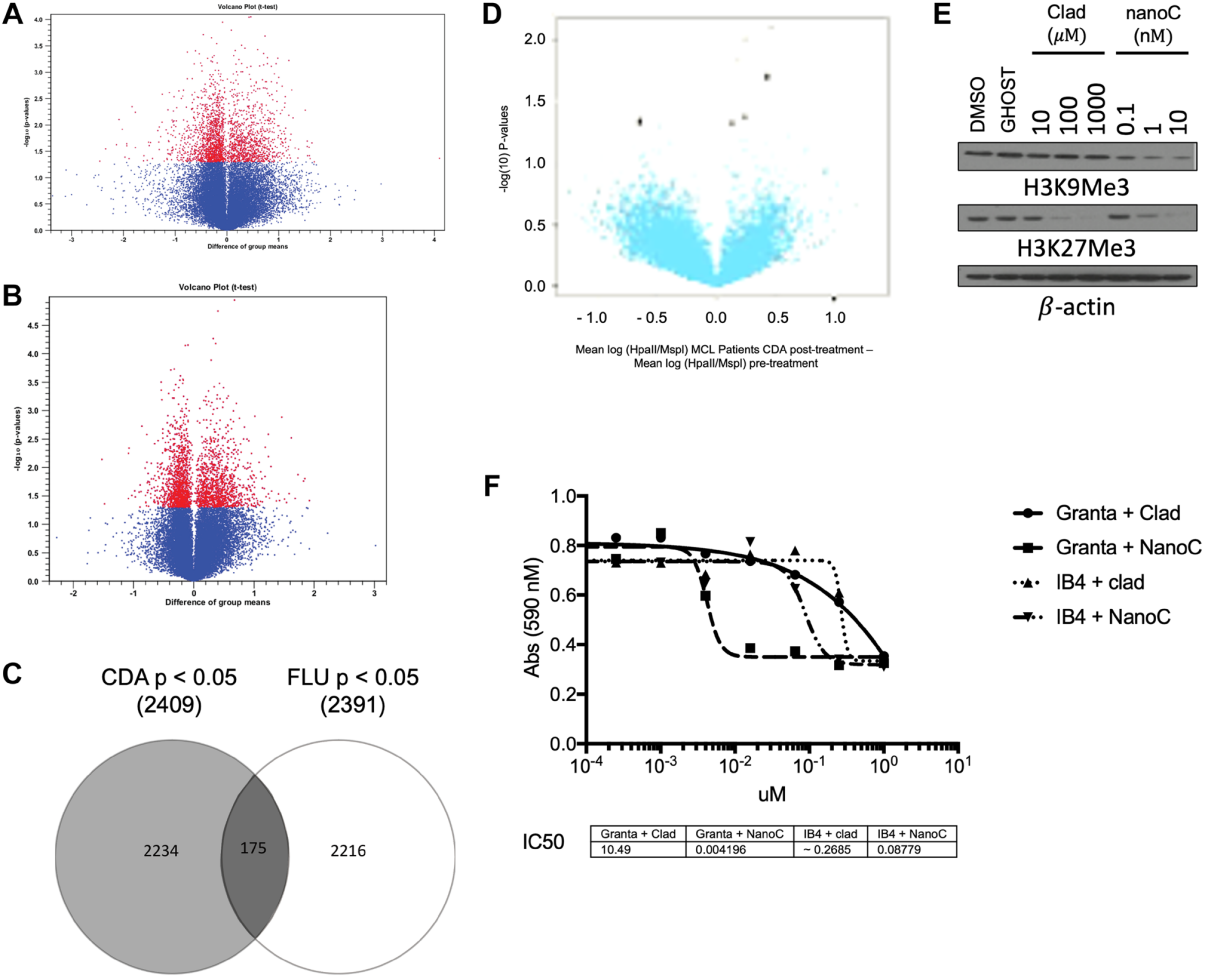
Cladribine has a unique epigenetic signature that affects DNA and histone methylation. (**A**) Plot of gene expression difference (X axis) vs. Significance (on Y axis) showing selected differentially expressed probes between CLL patients after treatment with fludarabine as compared to before it. Probe sets that were differentially methylated are marked in red (*p* < 0.05). Total 2 patients. (**B**) Plot of gene expression difference (X axis) vs. Significance (on Y axis) showing selected differentially expressed probes between MCL and CLL patients after treatment with cladribine as compared to before it. Probe sets that were differentially methylated are marked in red (*p* < 0.05). Total 3 patients (2 MCL). (**C**) A Venn diagram showing overlap between changed methylation state of genetic loci of fludarabine and cladribine (*p* < 0.05). (**D**) Volcano plot of methylation difference (X axis) vs. Significance (on Y axis) (using HELP) showing selected differentially methylated loci between patients after study treatment as compared to before it. Probe sets that were differentially methylated are marked in black (*p* < 0.05). Total 6 MCL patients. (**E**) Immunoblot of Granta 519 cells post 48 hours of treatment with increasing concentrations of cladribine (Clad) vs nanoliposome encapsulated cladribine (NanoC) assayed for H3K9Me3 and H3K27Me3. β-actin is loading control. (**F**) Cell viability by MTS assay of Granta 519 (Granta) and IB4 cells after 48 hours of treatment with varying doses of clad or NanoC. ^*^ *p* < 0.05 for Granta 519 cells. ^#^ *p* < 0.05 for IB4 cells.

*In vitro* gene profiling comparing changes in gene methylation and expression after treatment with fludarabine (Figure 1A) or cladribine (Figure 1B) in MCL patients showed that both cladribine and fludarabine altered methylation and gene expression patterns. Both drugs affected ~2400 genes, but only 175 of them were common between the two treatments (Figure 1C). *In vivo* HpaII tiny fragment enrichment by ligation mediated PCR (HELP) of cells from 6 MCL patients treated with cladribine revealed significant CpG hypomethylation at various loci (Figure 1D). In order to potentially improve the efficacy and toxicity profile of cladribine, we encapsulated it within anionic nanoliposomes (nanoclad). Treatment of Granta519 cells with nanoclad showed a more potent reduction in CpG hypomethylation at 100-fold lower doses than naked cladribine (Figure 1E). Additionally, *in vitro* treatment of Granta519 and IB4 cells showed increased potency of nanoclad over naked cladribine in reducing cell proliferation (Figure 1F).

### DUSP2 expression in MCL cells *in vitro* and *in vivo* after cladribine treatment

**Figure 2:**
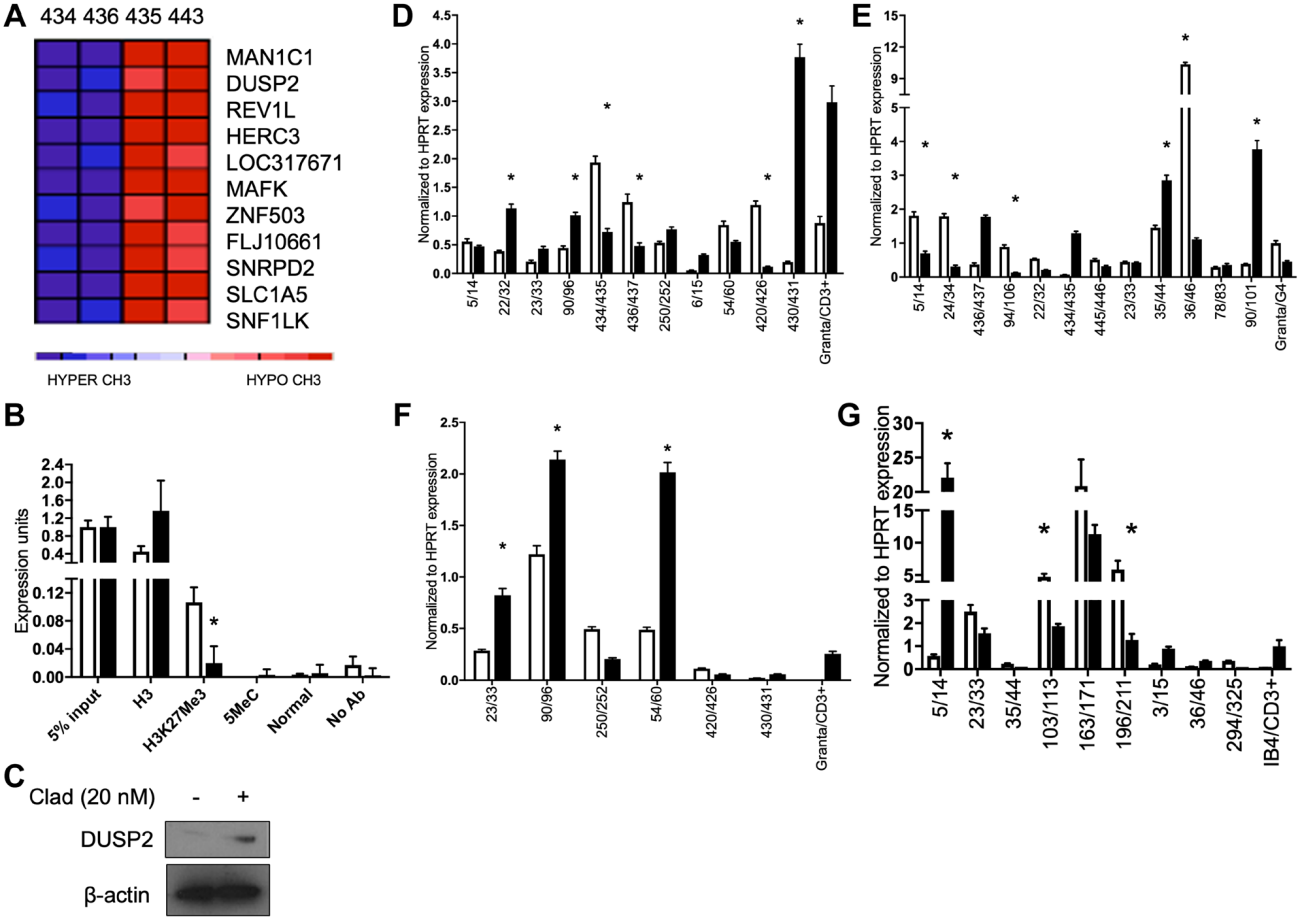
Cladribine derepresses the *DUSP2* gene and affects *DUSP1*, *TP53* and *CEBPB* expression. (**A**) Heatmap depicting a gene cluster containing *DUSP2* before (434, 436, blue) and after (435, 443, red) cladribine treatment of two MCL patients (434/435, 436/443). (**B**) ChIP assay showing decreases in H3K27Me3. (**C**) Immunoblot of Granta 519 cells for DUSP2 after treatment with 20 nM cladribine. β-actin is loading control. (**D**) qRT-PCR of MCL patient sample pairs before and 5 days after treatment with SCR for *DUSP2* transcript. (**E**) qRT-PCR of MCL patient sample pairs before and 5 days after treatment with SCR for *TP53* transcript. (**F**) qRT-PCR of MCL patient sample pairs before and 5 days after treatment with SCR for *DUSP1* transcript. (**G**) qRT-PCR of MCL patient sample pairs before and 5 days after treatment with SCR for *CEBPB* transcript. ^*^ *p* < 0.05. All statistics compared pre to post treatment.

Hierarchical clustering of HELP assay results from MCL patient samples comparing pre- and post-treatment with Pearson’s correlation showed a variety of significantly CpG hypomethylated genes including dual specific protein phosphatase 2 (*DUSP2)* (Figure 2A). *DUSP2* negatively regulates MAPK superfamily members, a commonly activated pathway in MCL [27]. Cladribine treated Granta 519 cells were assayed for epigenetic changes and transcriptional activation at the *DUSP2* promoter. Chromatin Immunoprecipitation (ChIP) assays on the *DUSP2* gene promoter in Granta 519 cells post treatment with 100 nM cladribine demonstrated decreased H3K27Me3 (Figure 2B) which correlated with increased protein expression (Figure 2C). In some MCL patients, *DUSP2* transcript was upregulated after treatment with cladribine (Figure 2D).

### Effects of epigenetic therapy on signaling pathways in MCL patients

We observed significant induction of individual genes in some patients (*DUSP1*, *p53*, *CEPB*) but with noticeable variability between patients. Patients upregulated different genes in the TP53, DUSP1 and CEPB pathways (Figure 2E, 2F and 2G respectively). This suggests that methylation inhibitors can affect different gene pathways *in vivo* in MCL patients.

### Cladribine and SCR therapy mildly increased apoptosis and signatures of ADCC in MCL

**Figure 3:**
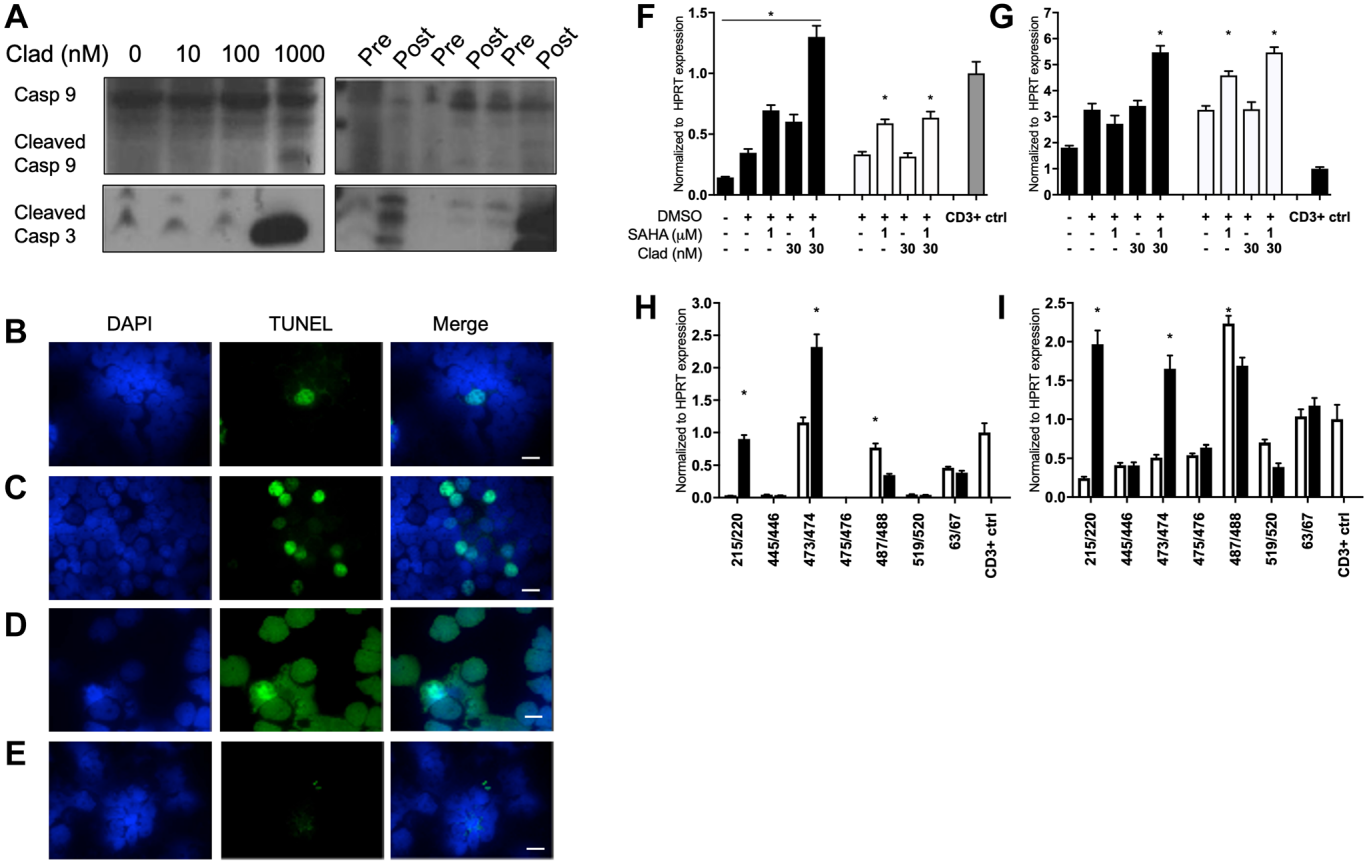
Cladribine and SCR therapy minorly increased apoptosis and signatures of ADCC in MCL. (**A**) (Left) Immunoblot of Granta 519 cells for cleaved caspase 3 and 9 after 48 hour treatment with increasing concentrations of cladribine. (Right) Three pairs of MCL patient samples assessed for caspase 9 and 3 levels before and after 5 days of SCR treatment. Full length caspase 9 was used as loading control. TUNEL staining for apoptosis of MCL patient (**B**) pre treatment and (**C**) post treatment with SCR. (**D**) 353 cells treated with DNAse as positive control. Scale bar is 10 μM. (Blue, DAPI; Green, TUNEL stain). (**E**) TUNEL staining of TPLL cells post treatment with cladribine, vorinostat and alemtuzumab as negative control [23]. (**F**) qRT-PCR of NKL cells for granzyme B (*GZMB*) expression after treatment with combination vorinostat and cladribine at increasing doses for 48 (black) or 72 (white) hours. (**G**) qRT-PCR of NKL cells for perforin (*PRF1*) expression after treatment with combination vorinostat and cladribine at increasing doses for 48 (black) or 72 (white) hours. (**H**) qRT-PCR of MCL patient sample pairs before and 5 days after treatment with SCR for *GZMB* transcript. (**I**) qRT-PCR of MCL patient sample pairs before and 5 days after treatment with SCR for *PRF1* transcript. ^*^ *p* < 0.05. NKL statistics are compared to DMSO treatment, and patient statistics are based on pre vs post treatment comparison. CD3 positive PBMCs were positive control.

Previous studies have shown that cladribine and vorinostat alone have limited antitumor activity in MCL [25, 28], and only with the addition of rituximab are dramatic antitumor effects observed. In contrast to what we have seen in T-PLL cells [24] we observe evidence of apoptosis in Grant 519 cells and MCL patient cells treated with epigenetic therapy. In Granta519 cells, caspase-9 and -3 cleavage was observed with high-dose cladribine indicating that cladribine can induce apoptosis in MCL as a single agent (Figure 3A, left). There were inconsistent changes in caspase-9 cleavage in cladribine and vorinostat treated MCL patients. However, caspase-3 cleavage was consistently increased in patient cells after treatment (Figure 3A, right). MCL patient cells treated with SCR showed increased moderate amount of apoptosis by TUNEL assay (Figure 3B–3E). However, *in vitro* apoptosis did not correlate with the sharp decline in leukemic MCL patient WBC counts after SCR treatment (Supplementary Figure 1), so we investigated ADCC as a potential other mechanism of cytotoxicity.

To determine whether epigenetic therapy could affect ADCC, NKL cells, an NK cell line, were treated with varying combinations of cladribine and vorinostat and showed increased expression of both granzyme B and perforin as compared to either treatment alone (Figure 3F, 3G). In MCL patient samples we observed variability between patients for granzyme B and perforin expression, with increased expression not consistent between all patients (Figure 3H, 3I). A recent report demonstrated that epigenetic therapy could potentially reverse epigenetic silencing of CXCL9 and 10 chemokines by the polycomb repressor and overcome resistance to immunotherapy [29]. Notably, among the 5 MCL patient specimens evaluated before and after SCR treatment, CXCL9 expression increased in one patient, whereas the other patients did not exhibit significant increases in expression (Supplementary Figure 2). CXCL10 expression was not detected in any patient sample (data not shown).

### Mechanisms of MCL treatment resistance

**Figure 4:**
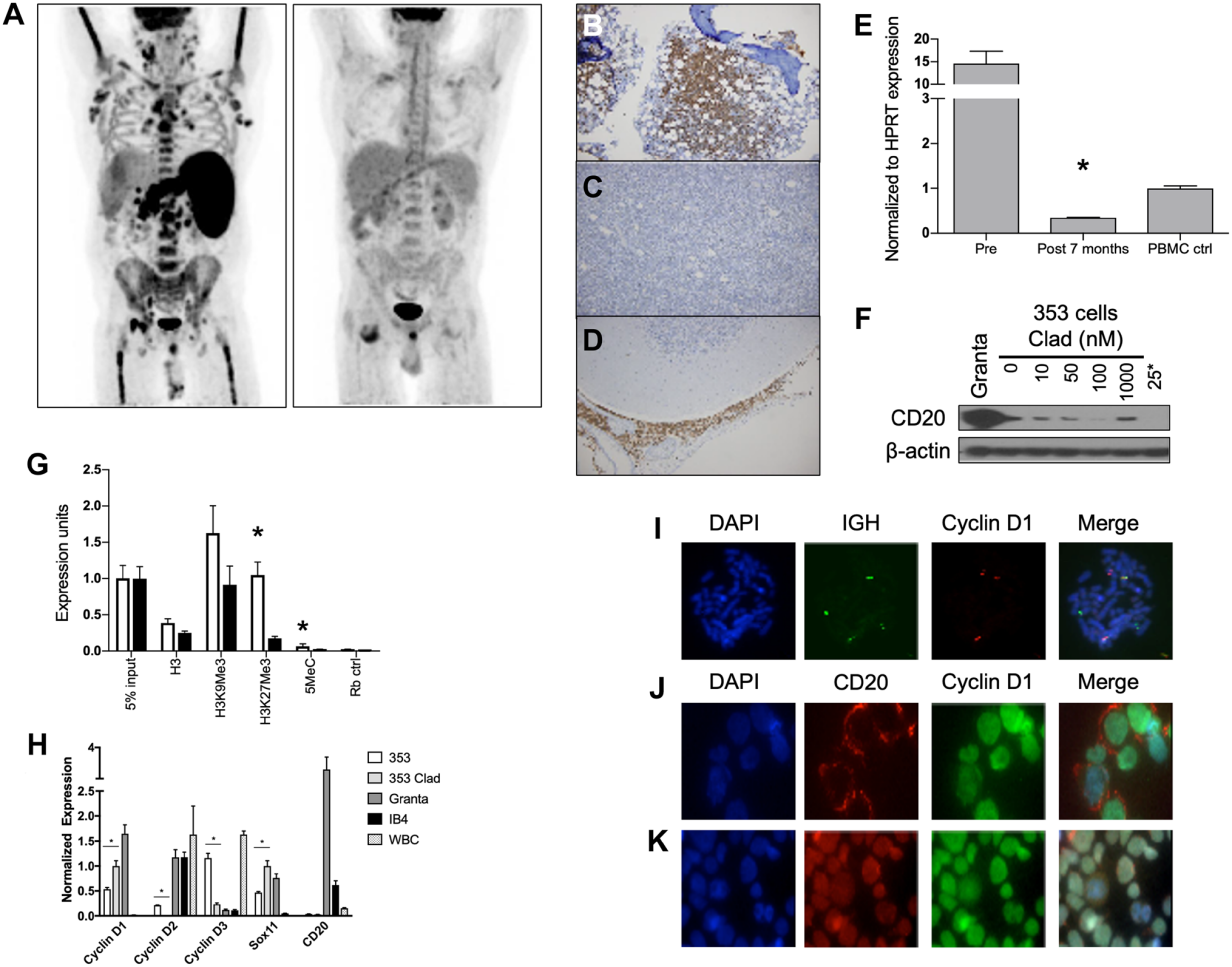
Mechanisms of SCR resistance and characterization of the CD20 negative MCL cell lines 353. (**A**) PET scans of MCL patient from case study pre (Left) and post (Right) 2 cycles of treatment with SCR. (**B**) Immunohistochemistry of diagnostic bone marrow biopsy showing infiltration of CD20 positive tumor cells (brown). (**C**) Immunohistochemistry of mediastinal lymph node biopsy at time of SCR resistant relapse showing infiltration with cells but lack of CD20 staining (brown). (**D**) Immunohistochemistry of cerebellum at autopsy showing CD20 positive (brown) cells. (**E**) qRT-PCR of CD20 (*MS4A1*) expression in cells taken prior to treatment initiation and at time of resistant relapse. (**F**) ChIP assay of CD20 promoter pre (white) and 3 months post (black) treatment with SCR for total Histone 3 (H3), H3K9Me3, H3K27Me3, methylated cytosine (5MeC) and random rabbit antibody control. (**G**) Immunoblot for CD20 in cells cultured from patient’s 7 month relapse (353 cells) post 48 hours of *in vitro* cladribine treatment. Granta is positive control. 25*- 353 cells cultured in 25 nM cladribine for 3 months. β-actin is loading control. (**H**) qRT-PCR for *CCND1, CCND2, CCND3, Sox11 and MS4A1* (CD20) levels in 353 (white), 48 hour 25 nM cladribine treated 353 (light grey), Granta 519 (dark grey), IB4 (black) and PBMCs (dotted). ^*^ *p* < 0.05 and compares 353 to cladribine treated 353. (**I**) FISH of 353 cells stained for DAPI (blue), *IgH* (green) and *CCND1* (red). White arrows point to translocation events. Immunohistochemistry for CD20 (red) and cyclin D1(green) in (**J**) Granta 519 cells and (**K**) 353 cells. Scale bars are 10 μM.

A 62-year-old male with newly diagnosed blastic leukemic MCL was enrolled to the SCR trial. Restaging PET/CT after 2 cycles of SCR therapy was consistent with metabolic CR with normalization of his WBC count and clearing of disease from bones, nodes and spleen (Figure 4A). His WBC remained low, so subsequent cycles of therapy were held. Ultimately he was removed from the study. Several months later, because of persistent dizziness and mental status changes, an MRI was performed and was consistent with leptomeningeal involvement of MCL confirmed by CSF analysis. SCR therapy was re-initiated, but neurologic deterioration continued, resulting in coma and eventually death. At autopsy, relapsed disseminated MCL was found in bone marrow, lymph nodes, and CNS. Initial bone marrow biopsy at diagnosis had been CD20+ MCL (Figure 4B) while relapsed systemic disease was CD20- (Figure 4C). The lymphoma in the CNS showed CD20+ MCL cells (Figure 4D). CD20 transcript levels at diagnosis compared to relapse decreased almost 40 fold (Figure 4E). These relapsed CD20- MCL cells were propagated in cell culture into a novel cell line, 353 cells. ChIP assay of the CD20 promoter pre vs. 3 months post treatment with SCR showed decreased H3K27Me3 and 5-methyl cytosine methylation after treatment (Figure 4F). These cells showed reduced CD20 protein expression compared to other MCL cell lines, such as Granta519, but remained trace CD20 positive in cell culture. Low-dose cladribine treatment for 48 hours minimally decreased CD20 levels while higher doses increased it. However, continuous treatment *in vitro* with low dose cladribine for 3 months completely abolished CD20 expression (Figure 4G). 353 cells were found to contain cyclinD1 and D2 levels lower than those of Granta519 but cyclin D3 levels that were higher. *Sox11* expression was also lower in 353 cells as compared to Granta519. Of note, cladribine treatment of 353 cells affected expression of all cyclin D proteins and *Sox11* (Figure 4H). 353 cells were confirmed to be MCL cells through fluorescence *in situ* hybridization (FISH) for the t(11;14) *IgH*-*CCND1* translocation (Figure 4I). 353 cells were confirmed to be CD20- through immunofluorescence (Figure 4J, 4K). Thus, this cell line recapitulated the phenotype and genotype of the blastic MCL patient from whom it was derived.

### Vorinostat, cladribine, rituximab were highly effective in treating blastic MCL

**Figure 5:**
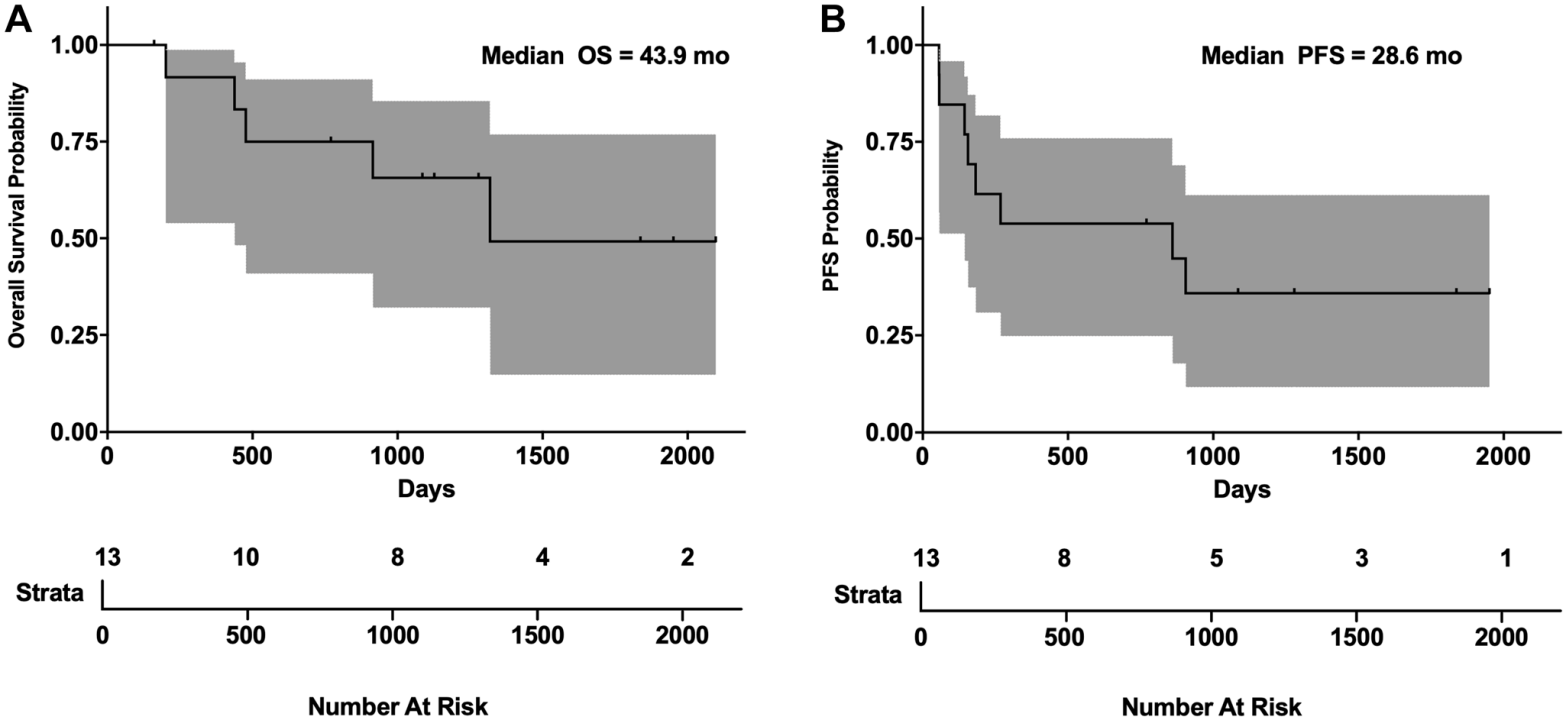
SCR PFS and OS in blastic MCL. (**A**) Overall survival and (**B**) progression free survival Kaplan-Meier plots of blastic MCL patients on SCR trial. Gray area represents 95% confidence interval. Ticks in graph represent censored patients lost to follow-up.

bMCL carries the worst prognosis within MCL subtypes [8]. A total of 13 bMCL (4 relapsed, 9 previously untreated) patients initiated treatment with the SCR regimen [22]. Results of patients treated off study were also reviewed retrospectively to augment numbers in this rare disease (10 patients on trial, 3 patients off trial). Due to rituximab intolerance (allergies, infusion reactions (*n* = 2)) or lack of efficacy, 4 patients were changed to ofatumumab, a potentially more potent fully human anti-CD20 antibody (Table 1). Patients received an average of 4.8 cycles of therapy. All patients were male Caucasian. The average age at diagnosis was 62 years old (Supplementary Table 1). The overall response rate (ORR) from initial SCR therapy was 11/13 (84%) with 5/13 (38%) attaining a CR. 3/13 (23%) progressed while on therapy and were switched to a different regimen. The median PFS was 28.6 months and OS was 43.4 months (Figure 5). Three patients who progressed on rituximab were changed to ofatumumab with 3/3 (100%) responding. Of 13 bMCL patients, all patients responded to therapy, with 12 patients meeting criteria for remission (CR, *n* = 6; PR, *n* = 6). Of those achieving CR, 5 remain in CR more than 5 years after diagnosis. One patient received radiation therapy for bone disease, failed a matched unrelated donor allogeneic stem cell transplant and after receiving SCR is now more than 9 years out in unmaintained CR. Another patient experienced a tumor lysis/anaphylactic reaction with his initial rituximab infusion requiring mechanical ventilation and a prolonged ICU stay. He was removed from study, and went on to receive ofatumumab with CR greater than 5 years. Two patients had their maintenance therapy interrupted with relapse.

**Table 1:** Patient treatment data and current status

Pt ID	Age	Initial/Relapse	Previous Tx	Chemo	# Cycles	Response	Maintenance	Maint. Length (mo)	Response	Other Tx	Dead (1) Alive (0)
1	63	Initial		SCR	6	PR	Ofatumumab	4	Progression	Bendamustine Radiation ESHAP GEM-OX Gemzar	1
2	79	Initial		SCR	4	PR	Ofatumumab + Sirolimus	32	Progression	N/A	0
3	66	Initial		SCR SCO	1 SCR 2 SCO	CR	Ofatumumab	28	CR		0
4	55	Initial		SCR	6	PR	Ofatumumab + radiation	6	CR	Radiation	0
5	79	Relapse	R-CHOP (PR) Bortez. (F) Benda + R (F)	SCR	2	Progression	N/A	N/A	N/A	ESHAP ESHAP Ofatumumab	0
6	57	Initial		SCR	6	CR	Ritixumab	8	CR		0
7	55	Relapse	Rituxan (F) Benda + R (PR) Radiation (PR)	SCR	6	CR	Rituximab	7	Relapse	Ibrutinib	0
8	67	Initial	Velcade (PR)	SCR SCR+V	3 2	PR then progression	Ofatumumab	4	Progression	Ofatumumab Ofa + Bendamustine	1
9	49	Initial		SCR CR	5 1	PR	Rituximab + Radiation	12	CR then relapase	Bendamustine/Velcade SCT	1
10	62	Initial		SCR	6	CR (Cycle 3)	Rituximab	48	CR		0
11	66	Initial		SCR	4	CR (Cycle 3)	N/A	N/A	N/A	Intrathecal chemotherapy Ara-C + Velcade	1
12	59	Relapse	Hyper-CVAD R-CHOP Velcade + R (Progression)	SCR	2	Progression (Cycle 2)	N/A	N/A	N/A	Bendamustine + Ofatumumab ESHAP Radiation SCT	1
13	52	Relapse	Rituxan CHOP ESHAP Bortez.	SCR SCO	3 3	PR	Ofatumumab	17	CR then relapse	SCO CR - then relapse Ofa + RT Ibrutinib - current (PR)	0

### The role of maintenance therapy in the treatment of MCL

Maintenance rituximab therapy has been shown to provide a small but significant benefit in MCL patients after induction treatment with R-CHOP or FCR [13]. Although the trial was not designed or powered to answer a maintenance question, we reviewed the charts of all MCL patients treated at PSU with maintenance rituximab/ofatumumab on the SCR trial (Supplementary Table 2). PSU patients were offered treatment with maintenance therapy until disease progression or unacceptable toxicity. PFS rates were high in both blastic and non-blastic patients, emphasizing the role for maintenance therapy in the treatment of MCL [30] for maintaining remission, particularly when SCR therapy is utilized (Supplementary Table 2). The Oregon Health Sciences University (OHSU) cohort of MCL patients on this multicenter trial were treated with 2 years of maintenance rituximab therapy and a drop in PFS was observed between 2 and 3 years post treatment [21], in contrast to the PSU cohort, where no drop in PFS between 2 and 3 years was observed.

### The cyclin D1 A/G polymorphism genotype correlates with blastic MCL phenotype and complete remission after treatment

**Figure 6:**
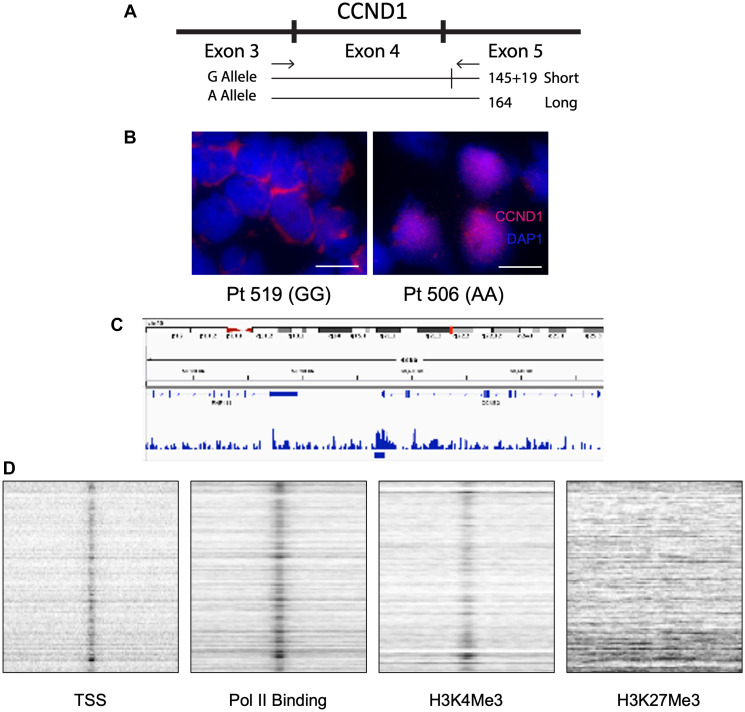
The G/A870 CCND1 polymorphism correlates with cyclin D1 cellular localization and differentiates blastic vs. non-blastic MCL. (**A**) Schematic of *CCND1* exons 3,4 and 5 and the splice site with the G allele that is not present with the A allele. (**B**) Immunohistochemistry staining of homozygous GG (Left) vs. homozygous AA (Right) MCL patient for cyclin D1 and DAPI. Scale bars are 10 μM. (**C**) Representative enrichment profile snapshot of genome browser from cyclin D1 ChIP-seq depicting sites of cyclin D1 binding to the genome. (**D**) Colocalization signals of cyclin D1 with transcription start sites (TSS), RNA polymerase II (Pol II), H3K4Me3 and lack of colocalization with H3K27Me3 from cyclin D1 ChIP-seq.

The polymorphism at the intron 4/exon 5 junction (G/A870) correlates with loss of cyclin D1 nuclear localization and differential transcript splicing (Figure 6A) [31]. Newly diagnosed MCL patients were genotyped for this polymorphism and their cyclin D1 localization assessed. Patients homozygous for the A870G mutation showed cytoplasmic cyclin D1 staining, whereas patients homozygous for the A allele showed nuclear localization (Figure 6B). To increase the number of patients, we included patients treated off-study with cladribine/rituximab-based regimens for a total of 42 newly diagnosed MCL patients. All genotyped patients’ response to treatment were then correlated with cyclin D1 localization and allele type (Table 2). The GG genotype had the best response and prognosis (CR: GG, 12/12; AG/GA, 13/17; AA, 6/13) with the presence of an A allele worsening treatment response. Interestingly, the AA genotype was associated with blastic MCL (blastic: AA, 12/13; AG/GA, 3/17) while GG patients were nonblastic (blastic: GG, 0/12). Because change in nuclear localization affected treatment response, we performed ChIP-seq assays for cyclin D1 binding and found as has been reported in nonlymphoid tissues it could bind specific DNA sequences in MCL cells [32–35] (Figure 6C). Additionally, ChIP-seq indicated cyclin D1 colocalized with transcription start sites, Pol II binding sites and histone 3 lysine 4 trimethylation (H3K4Me3) but not H3K27Me3 (Figure 6D). Although H3K4Me3 is a hallmark of replication origins [36], cyclin D1 did not co-localize with origins of DNA replication.

**Table 2:** G/A870 CCND1 polymorphism stratified by genotype

Genotype	Total patients with genotype	CR^*^	Blastic^**^
AA	13	6/13	12/13
AG/GA	17	13/17	3/17
GG	12	12/12	0/12

Number of patients achieving complete remission after SCR treatment (CR). Number of patients with blastic MCL (blastic). ^*^ *p* = 0.035 G allele is associated with CR, ^**^ *p* = 0.0001 A allele is associated with blastic MCL.

## DISCUSSION

Here, we describe results using cladribine based combination epigenetic and immunotherapy in MCL. A combined strategy using the SCR regimen was highly active, particularly in newly diagnosed MCL patients, with nearly all of these patients responding with > 80% achieving a CR including MRD negative CRs (BJH). Importantly, these remissions were durable, particularly when combined with maintenance therapy. We also explored the potential mechanism-of-action of the SCR therapy, resistance mechanisms and identify an important correlation with the G/A870 polymorphism, blastic disease and treatment response.

We found that some patients treated with SCR therapy demonstrated CpG hypomethylated *DUSP2*, removed repressive chromatin marks and an increase in transcriptional and protein expression. The CpG hypomethylation of *DUSP2* suggests that SCR may work through repression of MAPK signaling, as DUSP2 is responsible for deactivating MAPK pathway [37, 38]. However, not all patients showed this pattern, and the DUSP2 pathway should be further assessed for involvement in MCL progression. Similar findings were noted with *TP53*, *DUSP1* and *CEPB* transcripts though the precise roles of these genes is unclear.

Similarly to our results in TPLL [24], our SCR trial in MCL effectively demonstrated that epigenetic drugs can overcome resistance to rituximab. Importantly, we found that ofatumumab, in combination with epigenetic drugs, was able to overcome resistance to rituximab in some patients with resistant MCL. The SCR therapeutic approach should be tested with different tumor types and combined with PD1/PDL1 antibodies. This mechanism requires further study in regards to SCR in MCL and, further more, in immunooncology of epigenetic therapy and immunotherapy combination with both tumor type specificity and checkpoint directed monoclonal antibodies.

Unlike in the study of epigenetic therapy in T-PLL apoptosis appears to play a minor role in antitumor effects of SCR treatment in MCL [24]. We did appreciate increased ADCC, as evidenced by increases in and granzyme B levels after exposure to cladribine and vorinostat. However, other mechanisms remain to be studied. Recently, epigenetic silencing of CXCL9 and 10 cytokines by the polycomb repressor complex in solid tumors has been implicated in decreased T- and NK-cell infiltration into tumors. Decreased infiltration of killer cells into tumors increases in immunoprotection within tumors. Only epigenetic therapy inhibiting both DNA and histone methylation was successful in activating these silenced genes, validating our observations using cladribine. Of 5 MCL patient specimens evaluated before and after therapy, CXCL10 expression was not detected, whereas CXCL9 expression was detectable and was upregulated in one patient after SCR treatment. The patient-derived cell line 353 did not express CXCL9 nor CXCL10, and did not induce gene expression after exposure to cladribine or interferon. Together, our observations suggest that increased expression of CXCL9 correlates with therapy only in a minority of MCL patients.

In our current study, we identified two distinct resistance mechanisms to SCR therapy. The first mechanism involves harboring of CD20 positive disease in the CNS despite treatment with anti-CD20, likely due to the limited inability of rituximab to penetrate the blood brain barrier. This finding supports the notion that rituximab is essential for the antitumor effects of SCR and is in keeping with published data that cladribine or vorinostat monotherapy has some but limited single agent activity in MCL [28, 39]. The second mechanism of resistance involves the ability of malignant B cells to evade rituximab by downregulating expression of CD20, as evidenced by the presence of CD20- disease systemically. Loss of CD20 expression is a poor prognostic indicator for treatment with rituximab-based regimens, including SCR. In our experience, patients with CD20 negative B-cell malignancies have poor survival outcomes. In addition to the bMCL patient described in detail here, we have observed 3 cases of CD20 negative resistance in MCL, CLL, and MZL; as well as 3 cases of isolated CNS relapse in MCL, FL, and MZL. Future studies should evaluate monoclonal antibodies and ADCs against other B cell antigens, such as CD19 and CD22, that could help target this resistance mechanism.

The median OS of 43.4 months and PFS of 17.3 months for MCL patients with blastic disease treated with SCR therapy is one of the most important outcomes in this study. This is a significant improvement from the 14–24 months reported for other regimens [9]. Non-treatment naïve patients suffered from more relapses and did worse overall. These observations are similar to those obtained with classical MCL and TPLL and suggest prior chemotherapy may damage the epigenome and/or interfere with ADCC [19, 24]. Unlike in TPLL, four patients with bMCL achieved durable remissions without relapse that have lasted more than 5 years and continue to be disease free. Maintenance therapy with either rituximab or ofatumumab was capable of extending PFS in the majority of MCL patients regardless of MCL type. Collectively, our data demonstrates that epigenetic therapy in combination with monoclonal antibody immunotherapy is capable of producing durable remissions in both cMCL and bMCL.

An important finding was that the G/A870 *CCND1* polymorphism was a strong predictor of bMCL and poor treatment outcomes. In solid tumors, this polymorphism has shown conflicting findings regarding correlation with patient survival [31, 40–43]. The A allele correlates with more aggressive disease in cyclin D1 positive solid tumors [40, 44]. We found that patients homozygous for the A allele strongly correlated with the presence of bMCL, whereas G allele homozygosity strongly correlated with non-blastic disease and much better response to SCR therapy. Because the presence of the G and A allele have been determined to be about equal in MCL despite subtype [31], it can be inferred that the A allele is not a mutation that transforms cMCL into bMCL but rather that patients with the A allele have more aggressive disease. Testing for the G allele could be used to determine if MCL patients will respond well to SCR therapy and help to guide initial therapy, which is a pivotal decision point in MCL treatment, as well as requirement for and length of maintenance therapy.

This study has several limitations, including the relatively small sample size of bMCL patients. The use of maintenance rituximab was not mandated by the SCR clinical trial, but was left to the discretion of the treating physician and desire of the patient. One study looking at treatment in older patients with MCL showed an increase in OS with maintenance anti-CD20 therapy [13, 30]. The correlation of clinical factors such as MIPI score, Ki67 index, and cyclin D1 genotype with duration and type of maintenance therapy will require further study in larger controlled clinical trials. Although correlative studies identified important genes that were modified with epigenetic therapy, we were unable to definitively determine a transcript-based signature that could pinpoint the major effects of SCR on tumor cells, suggesting the importance of epigenetic effects on immune effector cells such as in ADCC. Observations regarding G/A870 allele frequency require testing in larger cohorts for further validation. However, the promising results of this SCR study in bMCL patients need validation in a larger randomized, prospective trial.

## MATERIALS AND METHODS

### Study design

This study analyzed only bMCL patients from an approved phase II clinical trial (NCT00764517) of SCR treatment of MCL performed under the supervision of two of the authors, Elliot Epner and Stephen Spurgeon at PSHMC and OHSU respectively. Due to the rarity of blastic MCL patients, only 13 patients were assessed in the prospective part of this study, with the remainder treated with SCR at PSU identified by retrospective chart review (PSU IRB 2000-186). Diagnosis and staging of classical and bMCL was made based on clinical presentation, immunophenotype, laboratory values and PET scan positivity. Blastic MCL diagnoses were also based on characteristic morphology and Ki67 staining indices of >30%. [45] Cheson criteria were used for determination of remission status. Complete remission was defined as undetectable disease by morphology and resolution of splenomegaly/lymphadenopathy confirmed by physical examination and/or computed tomography (CT) or PET scanning [46, 47].

Molecular remission was defined as flow cytometry negative for minimal residual disease and no detectable clone by cyclin D1 qRT-PCR analysis. Partial remissions were defined as 50% or more decrease in lymph nodes masses and in circulating tumor cell counts but lack of normalization of other complete remission criteria. Patients were treated until they achieved remission, met criteria for removal from the study/withdrew from the study or died. The clinical end points of this study were response rate, progression free survival, and overall survival. The objective of this study was to determine whether combined epigenetic and immunotherapies have efficacy and durable remissions in bMCL. One cycle of therapy was 28 days and defined as follows: rituximab given at 375 mg/m^2^ intravenously on days 3, 10, 17 and 24 on cycle 1 and then on day 3 for cycles 2–6; cladribine given at 5 mg/m^2^ on days 1 through 5; vorinostat orally at 400 mg on days 1 through 14. Maintenance rituximab was dosed at 375 mg/m^2^/1000 mg every 2–3 months. Clinical data collected were white blood cell counts, molecular and flow cytometric analyses of the blood and bone marrow and PET scans. Blood was collected before initiating therapy and at days 3 and 5 after therapy.

### Cell lines

Granta 519 is an MCL cell line that has been previously described and confirmed more recently [48, 49]. 353 cells were isolated from a blastic MCL patient on treatment with SCR therapy. It was isolated through continuous culture of PBMC isolated white blood cells. It is further characterized in this article. IB4 is a lymphoblastoid B-cell line derived from infection of neonatal B-lymphocytes with the B95.8 EBV virus [50]. Jurkat are T-cells derived from a 14 year old boy with acute lymphoblastic leukemia [51]. All cell lines were grown in RPMI-1640 with 10% FBS liquid media culture at concentrations not exceeding 1 million cells/mL.

### Antibodies

Anti-Cyclin D1 antibody [SP4] (Immunoblot) (abcam, ab16663), Anti-β Tubulin Antibody (H-235) (Santa Cruz sc-9104), Anti-H3K27Me3 (Millipore 05-1951), Anti-H3K9Me3 (Millipore 07-523), Anti-H3K27Me2 D18C8 (Cell Signaling #9728), Anti-H3k9Me2 (Millipore 07-521), Anti-β-actin (Millipore MAB1501), Anti-DUSP2 C-20 (Santa Cruz sc-1620-R), Anti-Caspase 3 (Cell Signaling #9662), Anti-Caspase 9 (Cell Signaling #9501), Anti-Cyclin D1 (IHC) (Santa Cruz sc-8392), Anti-CD20 (Santa Cruz sc-19990), normal rabbit antibody (Santa Cruz Biotechnology sc-2345), histone H3 positive control antibody (Cell Signaling #9715).

### Nanoliposomal production and formulation

Cladribine was loaded into anionic liposomes containing 1,2-distearoyl-sn-glycero-3-phosphocholine, 1,2-dioleoyl-sn-glycero-3-phosphoethanolamine, PEG (2000)-1,2-distearoyl-sn-glycero-3-phosphoethanolamine, and dihexadecyl phosphate at a 4:3:2:1 ratio. Nanoscale liposomes were formed by thin film rehydration foollowed by side-extrusion and sonication. Cladribine-loaded nanoliposomes were characterized as 90+/-8 nM with a zeta potential of -10mV. Ghost (control) liposomes of the same size, charge and lipid ratios did not contain any cladribine. Liposomes were purified from unincorporated cladribine on a sepharose CL-4B column. Concentrations of liposome encapsulated cladribine was assessed by LC-MS/MS. Chromatography was carried out on an I-class Acquity (Waters) chromatography unit with a C18 column. Multiple reaction monitoring on a Waters TQ-S was used to detect cladribine (286 < 170) and the internal standard propranolol (260.2 < 116.1). Concentrations were determined from a calibration curve.

### MTS assay

As previously described [38], 1–2 × 10^6^/ml cells were cultured in the presence of cladribine, or nanoencapsulated cladribine or ghost liposomes for 24–48 h. 96 well plates served to hold cells at a concentration of 2.5 × 10^4^ cells/well in 100 μL of culture medium. 100 μL MTS reagent (Promega, G3582) was added to each well and incubated for 2–4 h per the manufacturer’s instructions. Absorbance was measured at 590 nm on a Synergy HT plate reader (Biotek).

### Quantitative RT-PCR

White blood cells were isolated by gradient separation [23] and lysed in TRIzol (Ambion) per the manufacturer’s instructions. All RNA was treated with RQ1 ribonuclease-free deoxyribonuclease (DNase) (Promega) to remove DNA contamination. Samples were then reverse-transcribed, and qRT-PCR was carried out using SYBR Green (Qiagen) and BLAST designed primers (IDT). Primer sequences can be found in Supplementary Table 3.

### Immunoblot analysis

Analysis was carried out as described previously with some modifications [39]. Briefly, cells were lysed in RIPA buffer (Sigma, R0278) with 1:100 protease inhibitor (Sigma, P8340) and phosphatase inhibitor cocktail 2 (Sigma, P5726) and concentrations determined by BCA analysis (Thermo, 23225). 50 μg of protein was loaded on 10% precast Novex^®^ gels (Life Technologies) and run in the Xcell SureLock system (Life Technologies). Blots were blocked in either 5% BSA or non-fat dry milk for 1 hour prior to incubation overnight with the appropriate antibody. Signal was detected using anti-mouse HRP-conjugated secondary antibody (Cell Signaling, 7076) and developed on film (Kodak).

### Immunohistochemistry

Immunohistochemistry was performed as per the manufacturer’s instructions and with their reagents (Santa Cruz Biotechnologies).

### Fluorescence *in situ* hybridization (FISH)

FISH was performed as previously described [52]. Cells were fixed using 4% paraformaldehyde, methanol/acetic acid (3:1), and methanol/acidic acid, followed by paraformaldehyde with similar results. *CCND1* probes from chromosome 11 [53] were labeled by nick translation [52]. Hoechst dye was used as nuclear stain.

### ChIP assays

ChIP assays were carried out as described previously [24]. Briefly, frozen patient cells were suspended in phosphate-buffered saline (PBS) and fixed in 0.4% formaldehyde for 10-min rocking at room temperature. 5 min of 500 ml 2.5M glycine followed by cold PBS wash was used to quench fixation. Cells were lysed in cold tris buffer with 1:100 protease inhibitor cocktail (Sigma P8340) and incubated on ice for 30 min. Sonication was used on lysates with a Misonix Microson XL (25% power) for six cycles of 30 s on, 30 s off on ice. Debris was cleared with a 10-min, 17,000 g centrifugation. 50 μL of chromatin was diluted to 500 ml in dilution buffer. 15 μL of Magna ChIP Protein A+G beads (Millipore) and 10 mg of target antibody was added. Mixtures were precipitated overnight at 4°C and washed with 750 ml of the following buffers, each with 5-min incubation at 4°C with rotation: low-salt buffer, high-salt buffer, LiCl buffer and twice with TE. Chromatin was eluted from beads in 100 ml of elution buffer and 1 ml of proteinase K (Qiagen) at 64°C overnight. DNA was purified by PCR Purification Kit (Qiagen). DNA was quantitated by qRT-PCR using QuantiTect SYBR Green PCR Kit (Qiagen) on a Bio-Rad C1000 real-time thermocycler. All ChIP assay PCRs were done in triplicate.

For ChIP-seq, analyses were performed using 1% formaldehyde-fixed cells as instructed for the Millipore ChIP assay kit (Cat. no. 17-295). Following immunoprecipitations, crosslinking was reversed and DNA was isolated as described [54]. Libraries prepared with the isolated ChIP DNA as well as libraries of sonicated genomic DNA were sequenced using paired-end 101 bp reads with TruSeq V3 chemistry on a Hiseq2000 sequencing system. Samples were trimmed of adapters using Trimmomatic Software and aligned to the human genome (hg19) using Burrows-Wheeler Aligner (BWA) software. The MACS peak calling program was used, [55] comparing reads from ChIP –seq experiment to genomic input reads from the same cell line, with default parameters, and a *p*-value = 1e-9. Screenshots of example genomic loci were found using the IGV genome browser [56]. Degrees of colocalization with genomic features and chromatin modifications were calculated using the web-based ColoWeb program (http://projects.insilico.us.com/ColoWeb/) comparing genomic features from lymphocytes preloaded in ColoWeb to BED files denoting the locations of ChIP-Seq peaks [57]. The Genomatix suite (https://www.genomatix.de/) was used for validation.

### CpG/DNA methylation analysis by HELP

Genomic DNA was isolated, assayed and analyzed by comparative isoschizomer profiling as previously described [58–60]. Briefly, genomic DNA was digested by methylcytosine-sensitive enzyme, Hpa II, and by a methylcytosine-insensitive enzyme, Msp I. Products were amplified by PCR optimized to amplify 200 and 2000 bp with preference for cytosine-phosphate-guanosine (CpG) dinucleotide regions. Each fraction was then labeled and hybridized to a human HG17 custom-designed oligonucleotide array (50-mers) covering 25,626 Hpa II amplifiable fragments (HAFs) located at gene promoters and imprinted regions across the genome [60]. HAFs are genomic fragments between two Hpa II sites within 200 to 2000 bp from one another. Each HAF on is represented by 15 probes. Samples were processed at the Roche-NimbleGen Service Laboratory. PCR fragment length bias was corrected by quantile normalization. Quality control and data analysis was performed as described by Thompson et al. [61].

### TUNEL assay

A 5 m in cytospin cycle at 500 rpm was used to attach MCL cells to glass slides. Cells were stained with the Click-iT TUNEL Alexa Fluor Imaging assay (Invitrogen C10245) per manufacturer’s instructions and imaged on an Olympus BX60 upright fluorescence microscope.

### Cell cycle analysis

Cells were pulse labeled with 20 μM EdU for 45 minutes prior to cell harvest. EdU staining was performed using the Click-iT EdU kit (Invitrogen, C10424) according to manufacturer’s protocol. DAPI or PI were used for DNA staining. BD LSR Fortessa cell analyzer with FACS Diva software was used for cell cycle analyses.

### Statistical analyses

All significant *P* values were derived from two-tailed Student’s *t* tests with a cutoff of *P* < 0.05 and can be found in Supplementary Table 4. All experiments were repeated at least twice. Survival times were calculated from the start of SCR therapy. Standard Kaplan-Meier methodology was used to create survival curves.

## SUPPLEMENTARY MATERIALS


