# Supplementary material for: Combined epigenetic and immunotherapy for blastic and classical mantle cell lymphoma
Source: Oncotarget. 2022 Aug 16;13:986–1002. doi: 10.18632/oncotarget.28258 (PMC9450988; doi:10.18632/oncotarget.28258)
Supplement: Supplementary file 1 [file oncotarget-13-28258-s001.pdf]

Combined epigenetic and immunotherapy for blastic and classical mantle cell lymphoma

SUPPLEMENTARY MATERIALS

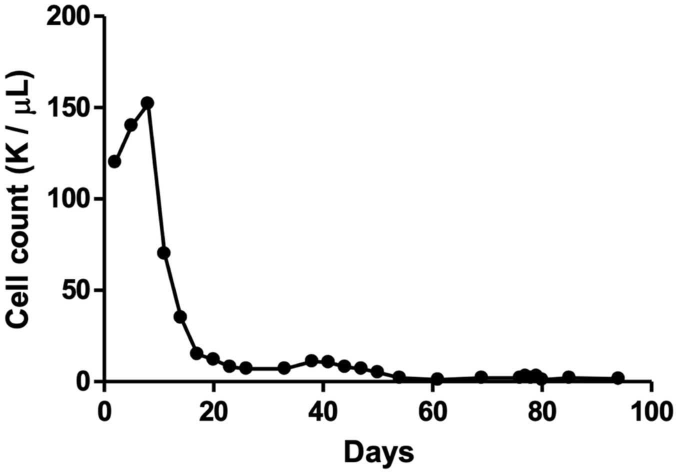

Supplementary Figure 1: Representative falling MCL patient WBC count during SCR therapy. Y axis in thousands of cells per microliter. Graph is representative of all patients on trial who showed a similar rapid decrease in cell counts. A single plot is shown for simplicity.

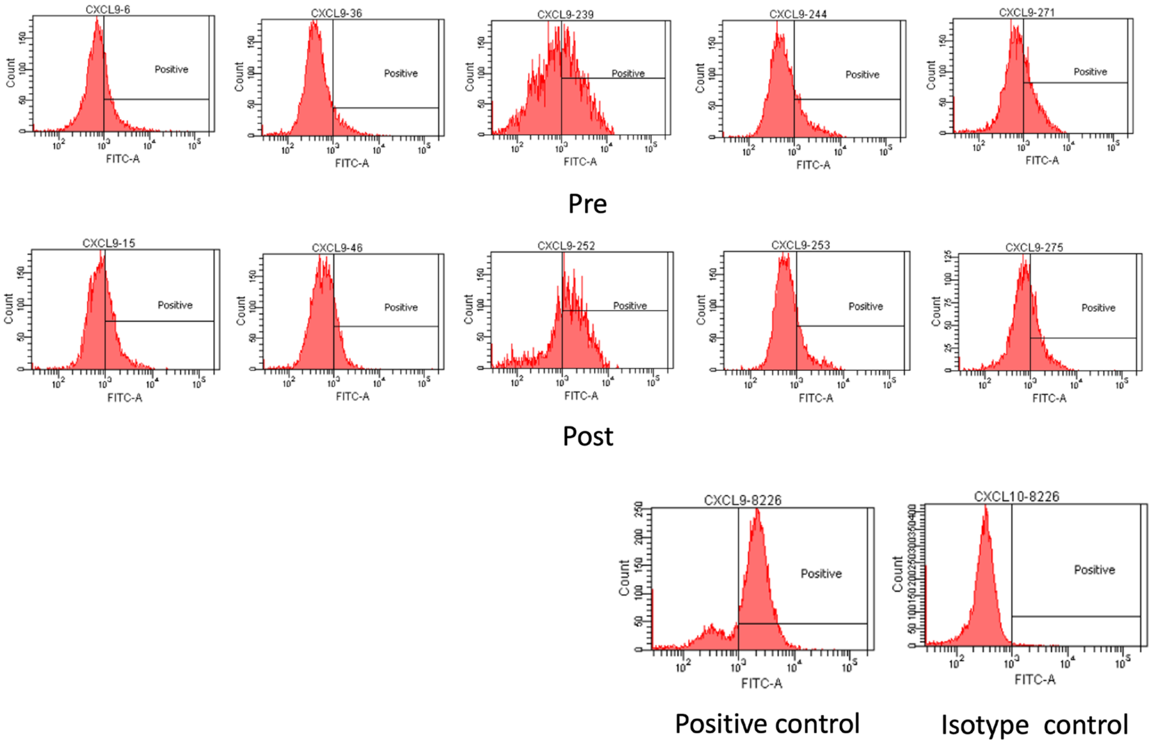

Supplementary Figure 2: CXCL9 expression level of patient samples.

Supplementary Table 1: Patient and disease characteristics

Patient	Age at First Dx	Relapse Yes = 1, No = 0	Stage	Blastic	Immunophenotype	Trans-location	Ki67 (%)	Blood	Marrow	Lymph	Spleen	Other involved site	WBC (K/µL)	HGB (g/dL)	PLT (K/µL)	PMN (K/µL)	Lymphs (K/µL)	LDH (U/L)	B2M (g/dL)
**1**	63	0	IV	1	CD5+CD20+	t(11;14)	40–50	1	1	1	1	-	6.9	10.8	69	5	1.2	772	6.74
**2**	79	0	IV	1	CD5+CD20+CD23+	Not done	20	1	1	1	1	Scrotum	13.3	11.9	147	2.9	9.7	986	11.4
**3**	66	0	IV	1	CD5+CD19+CD20+	Not done	5	1	1	1	1	-	12.7	12.6	227	8.7	2.2	1118	12.8
**4**	55	0	IV	1	CD5+CD19+ CD20+CD23+	t(11;14) TP53del 13q14del MYBdel ATMdel	30	1	1	1	1	-	12.07	13.2	152	3.26	7.85	605	4.08
**5**	79	1	IV	1	CD5+CD20+	t(11;14)	15–20	1	1	1	1	Pleura	13.7	11.6	357	6.7	6	1579	7.4
**6**	57	0	IV	1	CD19+CD20+	t(11;14) P53del	N/A	1	1	1	1	Tonsils	18.1	10	110	3.96	13.69	626	3.93
**7**	55	1	IV	1	CD20+	Not done	20-30	0	1	1	1	Cavernous Sinus/CSF	20.3	13	398	17.23	0.51	760	2.72
**8**	67	0	IV	1	CD5+CD20+	t(11;14)	20-30	1	1	1	1	-	32.7	7.9	23	3.3	29.4	785	7.04
**9**	49	0	IV	1	CD5+CD20+ CD22+CD38+	t(11;14)	30-40	1	1	1	1	Tongue, Nasopharynx	10.4	14.6	192	8.1	0.6	626	3.54
**10**	62	0	IV	1	CD19+CD20+CD23	t(11;14)	5	1	1	1	1	-	34.9	11.1	144	4.5	30.1	418	4.14
**11**	66	0	IV	1	CD5+CD19+CD20+	t(11;14)	10	1	1	1	1	-	23.3	9	52	8.4	7	730	5.97
**12**	59	1	IV	1	CD5+CD19+ CD20+CD22+	Not done	N/A	1	1	1	1	Colon	4.1	14.9	140	2.1	1.2	1021	1.86
**13**	52	1	IV	1	CD5+CD19+ CD20+CD22+	t(11;14)	N/A	1	1	1	1		42.9	7.5	70	2.1	40.8	651	4.46

Supplementary Table 2: Blastic and non-blastic MCL patients treated with maintenance therapy with rituximab or ofatumumab

Patient	Disease	Response to SCR	Maintenance	Duration (Years)	Response
214	Non-blastic MCL	CR	Rituximab	0.88	CR
215	Non-blastic MCL	PR	Ofatumumab	1.20	RELAPSE
231	Non-blastic MCL	CR	Rituximab	1.80	RELAPSE
220	Non-blastic MCL	CR	Rituximab	1.95	CR
217	Non-blastic MCL	CR	Rituximab	2.01	CR
202	Non-blastic MCL	CR	Rituximab	2.36	RELAPSE
201	Non-blastic MCL	CR	Rituximab	3.12	CR
208	Non-blastic MCL	CR	Rituximab	4.06	CR
206	Non-blastic MCL	CR	Rituximab	4.76	CR
1	Blastic MCL	PR	Ofatumumab	0.31	PROGRESSION
2	Blastic MCL	PR	Ofatumumab	2.62	PROGRESSION
3	Blastic MCL	CR	Ofatumumab	2.34	CR
4	Blastic MCL	PR	Ofatumumab	0.50	CR
6	Blastic MCL	CR	Rituximab	0.71	CR
7	Blastic MCL	CR	Rituximab	0.59	RELAPSE
8	Blastic MCL	PR	Ofatumumab	0.35	PROGRESSION
9	Blastic MCL	PR	Rituximab	1.02	CR THEN RELAPSE
10	Blastic MCL	CR	Rituximab	4.05	RELAPSE
13	Blastic MCL	PR	Ofatumumab	1.49	CR THEN RELAPSE

Supplementary Table 3: Primer sequences

**Taqman primer/probe (Life Technologies)**	
DUSP1	Hs00610256_g1
DUSP2	Hs00358879_m1
p53	Hs01034249_m1
A/G polymorphism primers	C_744725_1
Cyclin D1	Hs00765553_m1
Cyclin D2	Hs00153380_m1
Cyclin D3	Hs00236949_m1
CD20	Hs00254780_m1
18S	Hs03928992_g1
Beta Actin	Hs99999903_m1
**RT QPCR Primers (IDT)**	
CEBPB Forward primer	CGC CGC CGC CTG CCT TTA AAT
CEBPB Reverse primer	TAC GCA GCA GCC AAG CAG TCC G
granzyme B forward primer	GAG AGC AAG GAG GAA ACA ACA
granzyme B reverse primer	AAG ATA AGC CAT GTA GGG GCG
perforin forward primer	AAGTGA TGT GAGTGGTGG CTG
perforin reverse primer	GAATCC CGT ATA GAG AAG CGG C
SOX11 Forward primer	CCA GGA CAG AAC CAC CTG ATG
SOX11 Reverse primer	GCC TTG GAA AAT ACA CAC TTG TTA AC
SOX11 FAM probe	TTT AGA GCA GTT TTC AGC ATG A

Supplementary Table 4: Significant *p*-values

**Figure 1F *p*-values**								
**Condition**	**Untreated**	**250 pM**	**1 nM**	**4 nM**	**16 nM**	**64 nM**	**250 nM**	**1 µM**
Granta: nanoclad vs. clad	ns	n.s.	n.s.	0.0058	0.0061	0.0016	0.0003	n.s.
IB4: nanoclad vs. clad	ns	n.s.	n.s.	n.s.	n.s.	n.s.	0.005	n.s.

***p*-values**									
**Figure 2B**		**Figure 2D**		**Figure 2E**		**Figure 2F**		**Figure 2G**	
**Condition**	**Granta/Granta clad**	**Patient**	***DUSP2***	**Patient**	***TP53***	**Patient**	***DUSP1***	**Patient**	***CEBPB***
H3	n.s	5/14	n.s.	5/14	0.001	23/33	0.0013	5/14	0.0005
H3K27Me3	0.0155	22/32	0.007	24/34	0.0001	90/96	0.0014	23/33	n.s.
5MeC	n.s.	23/33	n.s.	436/437	0.0001	250/252	n.s.	35/44	n.s.
		90/96	0.0008	94/106	n.s.	54/60	0.0001	103/113	0.0043
		434/435	0.0006	22/32	n.s.	420/426	n.s.	163/171	n.s.
		436/437	0.0064	434/435	0.0001	430/431	n.s.	196/211	0.03
		250/252	n.s.	445/446	n.s.			3/15	0.0021
		6/15	0.0001	23/33	n.s.			36/46	n.s.
		54/60	n.s.	35/44	0.001			294/325	n.s.
		420/426	0.0001	36/46	0.0001				
		430/431	0.0001	78/83	n.s.				
				90/101	0.0002				

p-values					
	**Figure 3F**	**Figure 3G**		**Figure 3H**	**Figure 3I**
**Condition**	***GZMB***	***PRF1***	**Patient**	***GZMB***	***PRF1***
SAHA 1 uM 2 days	0.0029	n.s.	215/220	0.0001	0.0006
Clad 30 nM 2 days	0.0178	n.s.	445/446	n.s.	n.s.
SAHA 1 uM+Clad 30 nM 2 days	0.0006	0.0031	473/474	0.0052	0.0029
SAHA 0.5 uM 3 days	0.0028	0.0042	475/476	n.s.	n.s.
Clad 15 nM 3 days	n.s.	n.s.	487/488	0.0023	n.s.
SAHA 0.5 uM+Clad 15 nM 3 days	0.0052	0.0009	519/520	n.s.	n.s.
			63/67	n.s.	n.s.

p-values					
**Figure 4E**		**Figure 4F**		**Figure 4H**	
	**Pre/Post**		**Pre/Post**		**353/353 clad**
CD20	0.0068	H3	n.s.	*CCND1*	0.0138
		H3K9Me3	n.s.	*CCND2*	0.0001
		H3K27Me3	0.0082	*CCND3*	0.0006
		5MeC	0.0017	*SOX11*	0.0081
				CD20	n.s.

p-values					
**Supplementary Figure 1**		**Supplementary Figure 3**		**Supplementary Figure 5**	
**condition**	**Nanoclad/nanoclad+nano C6**	**condition**	**353/Jurkat**	**condition**	**post clad**
64 pM	0.0112	10 nM	0.0268	H3K4me2	n.s.
3.2 nM	0.0294	100 nM	0.0008	H3K9me2	0.0001
16 nM	0.0166	1 uM	0.0002	H3K27me2	0.0006
80 nM	n.s.	10 uM	n.s.		
400 nM	n.s.	100 uM	n.s.		
2 μM	n.s.				
